# Unlocking the Mysteries, Bridging the Gap, and Unveiling the Multifaceted Potential of Stem Cell Therapy for Cardiac Tissue Regeneration: A Narrative Review of Current Literature, Ethical Challenges, and Future Perspectives

**DOI:** 10.7759/cureus.41533

**Published:** 2023-07-07

**Authors:** Muhammad Abubakar, Muhammad Faiq Masood, Izzah Javed, Hira Adil, Muhammad Ahmad Faraz, Rakshita Ramesh Bhat, Mahek Fatima, Ahmad M. Abdelkhalek, Barbara Buccilli, Saud Raza, Mohsin Hajjaj

**Affiliations:** 1 Department of Internal Medicine, Ameer-Ud-Din Medical College, Lahore General Hospital, Lahore, PAK; 2 Department of Internal Medicine, Siddique Sadiq Memorial Trust Hospital, Gujranwala, PAK; 3 Department of Gastroenterology, University Hospital Monklands, Airdrie, GBR; 4 Department of Community Medicine, Khyber Girls Medical College, Hayatabad, PAK; 5 Department of Forensic Medicine, Post Graduate Medical Institute, Lahore General Hospital, Lahore, PAK; 6 Department of Medical Oncology, Mangalore Institute of Oncology, Mangalore, IND; 7 Department of Internal Medicine, Bangalore Medical College and Research Institute, Bangalore, IND; 8 Department of Internal Medicine, Osmania Medical College, Hyderabad, IND; 9 Department of Internal Medicine, Zhejiang University, Zhejiang, CHN; 10 Department of Human Neuroscience, Sapienza University of Rome, Rome, ITA; 11 Department of Internal Medicine, Jinnah Hospital Lahore, Lahore, PAK

**Keywords:** tissue engineering, regeneration, pluripotent stem cells, mesenchymal stem cells, ischemic cardiac tissue, embryonic stem cells, differentiation, crispr-cas9, biomaterials

## Abstract

Revolutionary advancements in regenerative medicine have brought stem cell therapy to the forefront, offering promising prospects for the regeneration of ischemic cardiac tissue. Yet, its full efficacy, safety, and role in treating ischemic heart disease (IHD) remain limited. This literature review explores the intricate mechanisms underlying stem cell therapy. Furthermore, we unravel the innovative approaches employed to bolster stem cell survival, enhance differentiation, and seamlessly integrate them within the ischemic cardiac tissue microenvironment.

Our comprehensive analysis uncovers how stem cells enhance cell survival, promote angiogenesis, and modulate the immune response. Stem cell therapy harnesses a multifaceted mode of action, encompassing paracrine effects and direct cell replacement. As our review progresses, we underscore the imperative for standardized protocols, comprehensive preclinical and clinical studies, and careful regulatory considerations. Lastly, we explore the integration of tissue engineering and genetic modifications, envisioning a future where stem cell therapy reigns supreme in regenerative medicine.

## Introduction and background

Ischemic cardiac tissue is damaged due to a reduced blood and oxygen supply. New methods for inducing cardiac tissue regeneration are being developed to improve the outcomes of surgeries [1]. Stem cell therapy for tissue regeneration and repair is of paramount importance due to the staggering impact of cardiovascular diseases (CVDs) on global health [2]. While stem cell therapy shows tremendous potential, it is perplexing why it has yet to receive widespread approval and adoption by regulatory bodies and healthcare systems [3]. For stem cell therapy to be effectively integrated into clinical practice, it is essential to bridge this gap.

The efficacy, safety, and role of stem cell therapy for the regeneration and repair of ischemic cardiac tissue remain areas of intense research [4]. Preclinical and clinical studies have shown encouraging results, but further exploration is needed to establish evidence-based guidelines and ensure safe and effective therapeutic outcomes. Questions regarding the optimal cell type, delivery method, dosage, long-term effects, and patient selection criteria remain [5-7].

**Figure 1:**
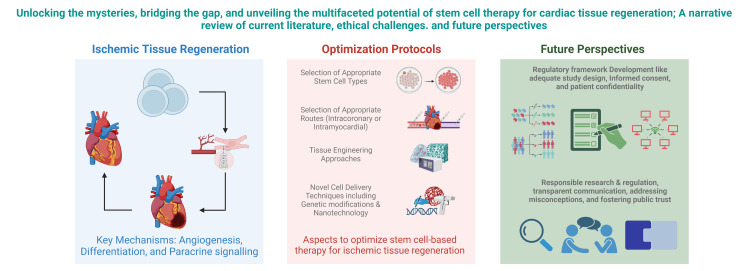
Gaps to Address in Stem Cell Therapy for Ischemic Tissue Regeneration and Repair Created with BioRender.com

Optimization protocols, novel delivery methods, tissue engineering techniques, and genetic modifications offer promising avenues to drive stem cell differentiation and enhance therapeutic efficacy. By fine-tuning the culture conditions, modifying gene expression, and utilizing biomaterial scaffolds, researchers can potentially guide stem cells toward cardiac lineage commitment, improving their integration, survival, and functional outcomes within the damaged cardiac tissue [8], as illustrated in Figure 1. However, despite these compelling hypotheses, the validation and comprehensive exploration of these strategies still need to be improved, representing a critical gap within stem cell therapy for ischemic cardiac tissue regeneration.

## Review

A significant global health concern

Ischemic heart disease (IHD) is a leading cause of mortality, contributing significantly to death rates in the US and worldwide. It is caused by the occlusion of the coronary arteries, which results in an oxygen supply-demand mismatch and plaque formation in the coronary artery lumen that obstructs the flow of blood. IHD impacts 126 million people across the globe (1,655 per 100,00), which can also be stated as 1.72% of the global population. Nine million lives have been taken by IHD worldwide, affecting men more often than women. The prevalence of IHD globally is expected to rise to 1,845 by 2030, necessitating novel techniques to improve patient outcomes [9]. In the US, 1.5 million cases related to myocardial infarction (MI) are recorded yearly, with an annual incidence rate of 600 cases per 100,000 persons. The incidence remains high and a global health concern [10,11].

Atherosclerosis is a primary cause of IHD, caused by the accumulation of fatty deposits in the arteries. This deposition can narrow arteries and decrease blood flow to the heart muscle. Other causes include high cholesterol levels, diabetes, hypertension, tobacco use, and a familial predisposition to heart disease [10].

The current treatments for IHD mainly focus on managing symptoms and preventing further damage to the heart. The treatments include pharmacological and non-pharmacological interventions. Surgical interventions may be necessary [12].

Are existing management options enough?

A surgical procedure known as coronary artery bypass grafting (GABG) involves rerouting venous or arterial conduits around blockages in the coronary arteries caused by atherosclerosis. This bypass restores blood flow to the ischemic myocardium, improving its function and alleviating symptoms of angina. While CABG is the most frequently performed major surgical procedure, with almost 400,000 surgeries yearly, surgical trends have declined with the growing use of alternative treatments like medical management and percutaneous coronary intervention (PCI) [13]. Despite these treatment options, IHD remains a leading cause of death worldwide. Therefore, a need exists for new and innovative treatments to address the underlying causes of IHD. Stem cell therapy is a promising treatment that has the potential to restore the ischemic heart [14].

The science behind stem cell therapy and its potential for cardiac tissue regeneration and repair

Stem cells possess unique regenerative and differentiation properties to revitalize damaged cells and tissues in the heart. The aim is to substitute injured or dead cardiomyocytes with new, healthy, fully functional counterparts, improve blood flow, and restore heart function [14,15].

Stem cells are non-specialized cells that have the ability to differentiate into various cell lineages within the organism. Stem cells currently being explored for cardiac regenerative medicine are categorized into two major types: pluripotent stem cells (PSCs) and adult stem cells (ASCs) [16,17]. The effectiveness of stem cell therapy for cardiac regeneration depends on the cell population's choice for transplantation. Until recently, cardiac muscle damage was considered irreversible; however, research has demonstrated that cardiac tissue can be regenerated with the help of stem cell transplantation [18]. The clinical studies investigating stem cell therapy for ischemic tissue regeneration in individuals with acute or chronic IHD have been inconsistent in their results. Variations in patient selection or study designs might contribute to the observed discrepancies [19].

ASC subtypes to repair and regenerate the heart tissue include cells derived from bone marrow-derived cells (BMCs), including mesenchymal stem cells (MSCs), and resident cardiac stem cells (CSCs) [15]. BMCs hold a prominent position in clinical studies for IHD due to their accessibility, abundance, and plasticity [20]. CSCs are self-renewing cells found in the myocardium and induced PSCs (iPSCs) are generated from ASCs. Both can transform into multiple cell lineages, including cardiomyocytes, making them promising candidates for cardiac tissue regeneration [15,21].

Types of stem cells used to repair, regenerate, and restore ischemic cardiac tissue

In this context, various stem cells have been employed, including MSCs, PSCs, and cardiac progenitor cells (CPCs). These diverse stem cell populations offer distinct regenerative properties and therapeutic potential for repairing damaged cardiac tissue.

Pluripotent Stem Cells (PSCs): Induced and Embryonic Variants

PSCs are currently being explored for cardiac regenerative medicine. PSCs can be obtained from two main sources: embryonic stem cells (ESCs) and iPSCs [22]. PSCs can differentiate into cardiomyocytes, the cells responsible for heart muscle contraction, making them an attractive candidate for cardiac tissue regeneration [23]. ESCs are sourced from the inner cell mass of an early-stage mammalian embryo during its initial stages of development [24]. iPSCs, conversely, are reprogrammed adult specialized cells using genetic reprogramming techniques [25]. ESC therapy holds great potential in regenerating and repairing ischemic cardiac tissue. ESC therapy involves transplanting PSCs into the damaged heart tissue to promote the repair response of diseased or injured tissue. ESCs can differentiate into multiple types of cells, including cardiomyocytes, and replace damaged heart tissue [26].

Adult Stem Cells, Bone Marrow-Derived Cells, Cardiac Stem Cells, and Mesenchymal Stem Cells

ASCs live within specific tissues that are differentiated while remaining undifferentiated and have the ability to regenerate or give rise to new cells that can restore damaged or dead tissue [27]. However, they are more limited in their potential compared to PSCs due to their sparseness in the native tissues, making it challenging to investigate and extract for research [27]. Finally, investigators have achieved notable advancements in exploring stem cell therapy for mending the heart, particularly focusing on CPCs. They comprise cardio-spheres (CSPs), their derived cells, and c-kit-positive CSCs [28].

Mechanisms of stem cell therapy in ischemic cardiac tissue regeneration and repair

Stem cells exert their beneficial effects on cardiac tissue rejuvenation and restoration through various mechanisms, encompassing angiogenesis, paracrine communication, and cellular differentiation.

Angiogenesis: Formation of New Blood Vessels

Recent studies have shown that stem cells promote angiogenesis and accelerate cell-based cardiac repair, particularly through the stimulation of endothelial cells. CSCs obtained from postnatal cardiac tissue have been studied as a potential means to promote the repair and regeneration of heart tissue through direct interaction with the affected area and indirect stimulation of endogenous myocardial cells [29].

Paracrine Signaling, Including Secretion of Growth Factors and Cytokines

Stem cell therapy involves the release of various factors that promote tissue repair and regeneration, known as paracrine signaling [30]. Human PSCs have been differentiated into cardiomyocytes that secrete paracrine factors that promote angiogenesis and reduce inflammation [31]. MSCs possess the capacity to undergo differentiation into cardiomyocytes or endothelial cells and apply their immunomodulatory properties, which include lowering inflammation and tissue damage by inhibiting neutrophil invasion [32,33].

Stem cells can release potent combinations of trophic factors, such as soluble substances and extracellular vesicles (EVs), which have lately emerged as a cell-to-cell communication method. These EVs are carriers for transferring chemicals between originator and recipient cells, altering their phenotypic and function. EVs can also trigger regeneration processes in wounded cells by regulating key cellular processes such as proliferation, angiogenesis, oxidative stress, inflammation, and immunological tolerance. These pro-regenerative actions of EVs are achieved by regulating key cellular processes such as proliferation, angiogenesis, oxidative stress, inflammation, and immunological tolerance [34,35].

In the context of regenerative therapy, it has been proposed that the beneficial effects of MSC therapy are mediated through paracrine signaling [36]. In a systematic review, 234 different factors were identified directly released by MSCs using various techniques. The most commonly identified factors were vascular endothelial growth factor, stromal cell-derived factor-1, fibroblast growth factor-2, insulin-like growth factor, interleukin (IL)-6 and IL-8, transforming growth factor type beta, and hepatocyte growth factor [36].

MSCs serve as a reservoir of soluble factors, and the surface microvesicles they release can potentially transport miRNA and RNA to injured organs. Paracrine effects play a significant role in contributing to the favorable outcomes observed in numerous clinical trials utilizing various stem cells [37,38].

Stem Cell Differentiation Into Specific Cell Types: Cardiac Muscle Cells, Smooth Muscle Cells, and the Endothelium

**Figure 2:**
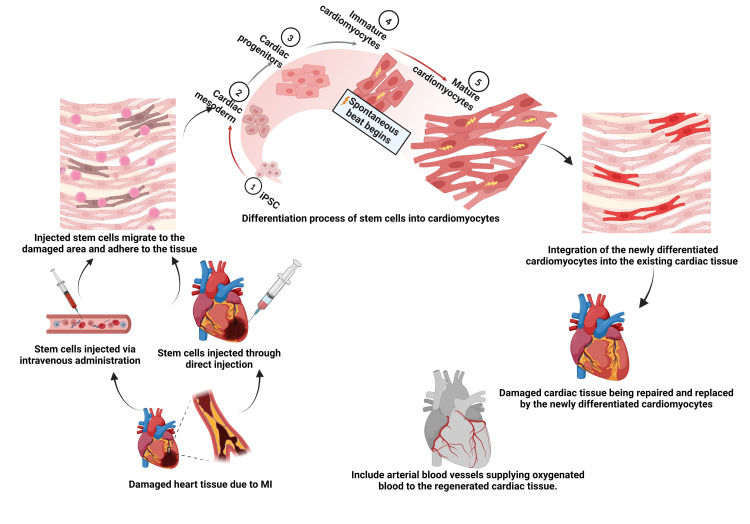
Cardiac Stem Cell Therapy (Decellularization and Recellularization of Whole Organs) Created with BioRender.com MI: myocardial infarction, iPSC: induced pluripotent stem cells

The final mechanism underlying stem cell therapy entails the differentiation into various cardiac cell types and replacing damaged or lost cells in the heart, as illustrated in Figure 2. Human PSCs have been differentiated into cardiomyocytes and transplanted into animal models, leading to improved cardiac function [31,39]. Overexpression of certain transcription factors has been shown to reprogram mouse fibroblasts into cardiomyocyte-like cells, potentially providing a novel source of cells for cardiac regeneration. However, new issues may arise from this approach, but if these findings are reproducible, they could provide a new therapy for generating de novo cardiac myocytes in vivo [40].

Stem cell differentiation into myocardial and endothelial cells is a crucial step toward achieving the full potential of stem cell therapy for CVDs [41]. Human iPSCs (hiPSCs) can be transformed into diverse cell lineages such as endothelial and smooth muscle cells (SMCs), according to various studies. The proportion of endothelial cells in cultures derived from hiPSCs was greater compared to cultures derived from ESCs, whereas the number of SMCs was lower in the former cultures than the latter ones [41-43]. hiPSCs hold promise as an advantageous cell reservoir for cardiac tissue engineering and heart repair through cell-based therapies. Under controlled laboratory conditions, these cells can undergo amplification and directed differentiation, giving rise to a broad range of cardiovascular cell lineages, encompassing cardiomyocytes [44].

Immunomodulatory Effects of Stem Cells

Recently, there has been a surge in research dedicated to unraveling the intricate interplay between stem cells and the immune system. Notably, MSCs have been shown to inhibit the activation and proliferation of different immunological cellular subsets, including T-cells, dendritic cells, and natural killer cells [37]. By doing so, they can attenuate the detrimental immune response associated with CVDs like MI and heart failure (HF).

The immunomodulatory effects of MSCs arise through dual mechanisms: the release of paracrine factors and direct interactions with other cells. Paracrine factors include hepatocyte growth factor, IL-1B, transforming growth factor-beta, prostaglandin E2, and indoleamine 2,3-dioxygenase. By acting on immune cells, these factors suppress their activity, block the production of pro-inflammatory cytokines, stimulate the secretion of anti-inflammatory cytokines, and foster the emergence of regulatory immune cell populations. This immunomodulatory environment created by stem cells can help reduce inflammation, limit tissue damage, and promote tissue repair in the cardiac microenvironment [37,45-48].

MSCs have also demonstrated the ability to interact with various immune cell populations, including macrophages, and modulate their polarization toward an anti-inflammatory phenotype. This novel mechanism of stem cell-mediated improvement in cardiac function, potentially through an IL-10-mediated transition from the infiltration of pro-inflammatory macrophages to anti-inflammatory macrophages at the site of infarct, validates the significance of IL-10 while augmenting the anti-inflammatory impact of such therapy in the process of cardiac regeneration [49].

Efficacy of stem cell therapy to repair, regenerate, and restore ischemic cardiac tissue

Building upon the immunomodulatory effects of stem cells, their efficacy in repairing and regenerating ischemic cardiac tissue has emerged as a promising approach, opening new avenues for improving patient outcomes in such diseases.

Evidence From Preclinical Studies (In Vitro and Animal Studies)

Bone marrow-derived MSCs have demonstrated a good safety profile and early effectiveness in enhancing regional contractility, improving quality of life, and reducing scar formation [50,51]. In rodent models of MI, some studies demonstrated that BMCs exhibit a more favorable survival pattern in improving cardiac function, reducing scar formation, and increasing angiogenesis [52]. Another study on the rats model found that the engrafted adult bone marrow containing MSCs can modulate the signaling of extracellular collagens and cytokines to prevent pathologic thinning of the ventricular scar and attenuate the compromised contractile function in the heart post-infarction [53]. In vitro studies have shown that these cells not only release paracrine factors but also cytokines to promote cell survival, angiogenesis, and tissue remodeling [54-56]. These studies underscore the regenerative capacity of stem cells in the structural and functional enhancement of ischemic myocardium.

Animal studies have provided evidence regarding the effectiveness of stem cell-mediated therapeutic interventions in ischemic cardiac tissue regeneration. An investigation was conducted to transplant cardiomyocytes derived from hiPSCs (hiPSC-CMs), into a heart attack model comprising non-human primates. The study unveiled the persistence and integration of transplanted cells into the host myocardium, leading to a substantial improvement in cardiac function [57].

In vitro investigations have yielded valuable understanding regarding the intricate mechanisms underlying stem cell therapy. A study demonstrated the expression of surface markers and the pluripotent capacity for multi-lineage differentiation exhibited by MSCs. Two extensively studied cell types include CSPs and c-kit expressing CSCs; which have shown positive outcomes in studies conducted in controlled laboratory environments, animal models, and initial clinical trials (Phase I) [28]. Results from a meta-analysis that involved merely large animals indicated safety and effectiveness in preserving left ventricular ejection fraction (LVEF) in cell therapy [58,59]. However, long-term safety assessments and plausible hazards entwined with stem cell therapy necessitate additional scrutiny and in-depth exploration.

Limitations are inherent in the existing preclinical investigations, including factors like the age of the animal subjects, the homogeneity of animals within a specific strain, and the absence of standardized protocols featuring consistent endpoints and outcome measures. To advance the clinical translation of stem cell-mediated therapeutic interventions, it is imperative to undertake extensive studies on a large scale [60,61].

Evidence From Studies in Humans, Including Randomized Controlled Trials and Observational Studies

Trials in human beings have been successful, with transplantation of MSCs after acute MI significantly increasing LVEF, showing a higher efficacy if done in the first week, according to a recent large meta-analysis [62].

Several clinical studies have demonstrated the therapeutic potential of PSCs in treating IHD. A clinical trial was done where a fibrin-based scaffold infused with a substantial population of 5-10 million PSC-cardiac precursor cells was grafted onto the epicardial surface of six patients with ischemic cardiomyopathy and comorbid congestive cardiac failure. Remarkably, the trial showed significant improvement in cardiac function. With the exception of the most recently enrolled patient, the remaining participants exhibited enhanced LVEF and symptomatology, alongside newfound contractility within the treated region. Nonetheless, the precise contribution of the fibrin patch versus the concomitant CABG procedure to the improved LVEF and symptoms remains unclear [23]. Furthermore, an additional study exhibited the beneficial effects of iPSC-derived cardiomyocytes on cardiac function and infarct size reduction in a comprehensive animal model featuring acute MI [63]. The technical possibility of creating CPCs derived from human ESCs for clinical use was demonstrated, along with evidence of their short and medium-term safety. These studies establish a solid foundation for conducting sufficiently large efficacy studies [64].

The BOOST-2 trial considered individuals receiving PCI following acute MI. The trial revealed that the introduction of self-derived BMCs into the coronary vasculature elicited a notable enhancement in LVEF and infarct size reduction, surpassing the outcomes observed in the control arm [65,66]. The REPAIR-AMI Trial was a randomized, controlled trial that also performed the same experiment in patients with acute MI and demonstrated similar efficacy of BMC therapy to improve LVEF compared to the control arm [67].

Factors Influencing Efficacy, Such as the Timing of Treatment, Type and Dose of Stem Cells Used, and Route of Administration

Despite these promising findings concerning stem cell therapy's efficacy, there are challenges to overcome. By default, concerns have been voiced regarding the inherent possibility of adverse effects. Notably, severe calcification after injecting BMCs in animal trials was highlighted [68,69]. Timing of stem cell injection is important, with a pronounced benefit if applied seven days after the cardiac event [58]. Long-term follow-up is another conundrum, as the first human trials using stem cell therapy to treat MI and HF occurred in 2009 and 2013, respectively [70,71]. Future pivotal inquiries remain regarding the key distinctions between exogenous progenitor and cardiac-derived cells.

Safety of stem cell therapy in repairing, regenerating, and restoring ischemic cardiac tissue

In addition to addressing the challenges surrounding stem cell therapy's efficacy, we must place an even greater emphasis on the safety of such procedures, which encompasses the potential hazards of the emergence of tumors, arrhythmogenic complications, immune rejection, and the like.

Tumorigenicity: The Ability of a Cell or Tissue to Form a Tumor

Teratoma formation refers to the development of neoplastic growths comprising cells originating from all three germ layers, while PSCs represent undifferentiated cells possessing the remarkable potential to undergo differentiation into any cellular lineage found throughout the body [22]. Compared to their embryonic counterparts, iPSCs are susceptible to a diverse array of factors that can promote oncogenic transformation. These factors encompass the integration of reprogramming vectors within the genomic structure, excessive expression of cancer-producing transcription factors, and an extensive hypomethylation profile similar to that observed in malignant tumors [72,73].

Teratomas should be considered while analyzing the danger of employing PSCs [74]. To diminish the potency of the cells and reduce the chance of tumor formation, it is preferable to differentiate ESC and iPSC into particular cell types in vitro. One way to address this is by stearoyl-CoA desaturase-1 targeting. However, current analytical techniques are not sensitive to detect the eradication of all PSCs [75]. Based on the theory of cancer stem cells, a minute fraction of tumor cells have the capacity to multiply on their own and meet the requirements for cancer stem cells. These stem cells are thought to regulate the perpetual regeneration of cells in the body and have the ability to multiply, disseminate cancer to secondary hosts, and cause cancer in their primary hosts [76].

Immunogenicity: Being Susceptible to Immune Recognition and Subsequent Destruction

Initially thought to be immune privileged, ESCs were later found susceptible to immune recognition [77]. Similarly, iPSCs are potential sources of autologous pluripotent cells. However, recent evidence indicates that even iPSCs derived from an individual's own cells may be susceptible to immune rejection [78]. Although PSCs may have immune modulation capabilities, successful therapy will require immune interventions as these cells are prone to rejection in their absence [79]. Achieving the right balance is crucial. The enhanced immune function would serve as a safeguard against tumor genesis and ensure the rejection-free acceptance of grafts. At the same time, diminished immune function would facilitate cell engraftment and increase the risk of tumor formation if the transplanted cells misbehave.

Arrhythmogenicity: The Risk of Developing Fatal Arrhythmias

Ventricular arrhythmias represent abnormal cardiac rhythmicity that has the propensity to culminate in sudden cardiac death [80]. Arrhythmias in the context of stem cell therapy for HF present a genuine safety concern [81]. The mechanisms behind arrhythmias include gap-junction imperfection, diminished cardiac excitability of transplanted cells, electrophysiological heterogeneity, enhanced automaticity, and problems with transplantation techniques [11].

Microvascular Occlusion: Compromised Flow of Blood Within the Microvasculature Bed

Impaired microvascular hemodynamics, encompassing the compromised perfusion within the microvasculature of cardiac tissue, raises substantial concerns regarding the safety profile of stem cell therapy [82]. The route through which stem cells are administered is a critical determinant of the likelihood of microvascular obstruction (MVO). For example, intracoronary infusion of stem cells quickly after acute MI may produce severe MVO, leading to myocardial inflammation and restricting cell retention and engraftment [83].

Strategies to mitigate the risks

Considering the paramount importance of ensuring the safety of stem cell therapy in repairing, regenerating, and restoring ischemic cardiac tissue, several strategies have been explored to mitigate the risks and enhance the safety profile. These include optimizing the cell selection and delivery route, improving cell retention and engraftment, genetic modification, developing novel delivery methods, and modulating the immune response. Researchers have developed techniques to differentiate PSCs into cardiomyocytes to address teratogenicity and gene editing to overcome immunogenicity by creating universal donor PSCs that could be transplanted into any patient without immune rejection [84].

The risk of arrhythmias can be reduced using a minimally invasive delivery method, such as intracoronary injection, and the type of stem cells used can also affect the risk of arrhythmias. For example, prevailing clinical evidence has demonstrated that BMCs exhibit a comparatively diminished risk of arrhythmias than skeletal muscle progenitor cells [81]. Table 1 summarizes such strategies.

**Table 1:** Strategies to Mitigate Risks in Stem Cell Therapy for Ischemic Myocardial Tissue Repair and Regeneration

Strategy	Description
Cell Selection	Careful selection of appropriate stem cell types based on their differentiation potential, survival rate, immunomodulatory properties, and ease of isolation. This strategy will address the issues of teratogenicity and immunogenicity.
Cell Purification	Removal of unwanted cell types and debris to ensure a more homogeneous population of stem cells.
Cell Characterization	Thorough characterization of stem cells to confirm their identity, purity, viability, and functional properties.
Preconditioning	Application of specific stimuli, such as hypoxia or bioactive molecules, to augment the therapeutic capacity and viability of grafted stem cells.
Genetic Modification	Genetic engineering techniques to modify stem cells with desirable traits, such as enhanced survival, differentiation, and paracrine signaling.
Delivery Methods	Optimization of delivery routes and techniques, including direct injection, intracoronary infusion, or tissue-engineered scaffolds. This will address the issues of arrhythmogenicity and microvascular occlusion.
Immunosuppression	Administration of immunosuppressive agents to modulate the immune response and prevent rejection of transplanted stem cells.

Challenges and limitations implicated in stem cell-based therapy

Alongside the strategies to mitigate risks and enhance the safety profile of stem cell therapy, it is essential to acknowledge the existing challenges and limitations that need to be overcome to ensure its effective translation into clinical practice.

Suboptimal Integration of Engrafted Stem Cells Into Functional Frameworks

Successful incorporation of engrafted stem cells into the existing cardiac tissue is critical for functional recovery [85]. Promoting proper alignment, electrical coupling, and structural incorporation of the engrafted cells with the host cardiac tissue is always challenging. Strategies like tissue engineering scaffolds, biomaterials, and electrical stimulation are being investigated to improve incorporation [86].

Poor Attainment of Functional Cardiomyocyte Differentiation in Transplanted Stem Cells

Ensuring the successful and targeted differentiation of transplanted stem cells into fully functional cardiomyocytes is crucial for cardiac tissue regeneration. Achieving efficient and controlled differentiation remains a challenge. Various novel approaches to induce cardiac protein expressions and gene manipulation, such as using specific growth factors, small molecules, or proteins, are underway to investigate the augmentation of the differentiation capacity of these cells toward the desired cardiomyocyte lineage [87].

Low Cell Survival and Viability

Ensuring the survival of transplanted stem cells is a major challenge in cardiac tissue regeneration. The harsh microenvironment, ischemia, and inflammation can lead to poor cell survival and limited therapeutic efficacy. Approaches aimed at augmenting cell survival encompass various strategies, such as priming through preconditioning methods involving the application of thermal stress, hypoxic conditions, and exposure to oxidant stress. Furthermore, the concurrent administration of complementary agents, including diminutive molecule inhibitors, exosomes, peptides, and miRNAs, serves as a means to bolster cell endurance. Additionally, genetic manipulation and the simultaneous transplantation of distinct cellular lineages are also employed strategies to fortify cell survival and viability [85].

Immune Rejection and Inflammatory Response Against Transplanted Stem Cells

The response triggered by transplanted stem cells can influence their survival, engraftment, and therapeutic outcomes. Immune rejection and immune-mediated damage to transplanted cells are significant limitations. Immunomodulatory approaches, such as immunosuppressive drugs, cell surface engineering, and co-transplantation with immunomodulatory cells, are being explored to mitigate immune responses [88]. Table 2summarizes the challenges faced by stem cell therapy with potential solutions to address.

**Table 2:** Challenges/Limitations of Stem Cell-Based Therapy to Repair and Regenerate Ischemic Myocardium With Potential Solutions and Future Perspectives

Challenge/ limitation	Description	Solutions (future perspectives)
Low cell survival and viability	The low survival rate of transplanted stem cells is due to the hostile microenvironment, limited nutrient supply, and immune response	Optimization of cell delivery methods; preconditioning of stem cells with co-administration of pro-survival factors or anti-apoptotic agents; and use of biomaterials and extracellular vesicles.
Immune response	Immune rejection and inflammatory response against transplanted stem cells, leading to cell death and limited therapeutic efficacy.	Co-administration of immunosuppressive agents to dampen the immune response; engineering of stem cells to evade immune recognition, such as through gene editing techniques (like CRISPR/Cas9 gene editing); modification of expression of major histocompatibility complex (MHC) molecules; and, encapsulation of stem cells within immunoprotective biomaterials or hydrogels to shield them from immune attack.
Inefficient differentiation	Challenges in directing stem cells to differentiate into functional cardiac cells and achieve desired cell phenotypes.	Identification of specific signaling pathways and growth factors that promote cardiac lineage commitment and differentiation; development of biomimetic scaffolds or 3D culture systems that mimic the cardiac microenvironment; and, genetic or epigenetic manipulation of stem cells to enhance their differentiation potential toward cardiac lineages.
Risk of tumorigenesis	Potential for transplanted stem cells to undergo uncontrolled growth and form tumors in the cardiac tissue.	Rigorous screening and characterization of stem cell populations to exclude cells with tumorigenic potential before transplantation; genetic modification of stem cells to inhibit tumor formation.
Risk of arrhythmogenicity	Low excitability because of imperfect gap junctions and small action potential duration (APD) of normal cardiac cells can lead to conduction blocks and ventricular arrhythmias, respectively	Genetic modification to control the extent of expression of these molecular cell-cell communication portals; develop ways to predict APD and synchronize before the transplantation of cells.
Limited engraftment	The Limited ability of transplanted stem cells to engraft and persist in the cardiac tissue, reducing long-term therapeutic effects.	Optimization of cell delivery methods and tissue engineering approaches to enhance engraftment and retention; genetic modification of stem cells to improve their adhesion properties for engraftment.
Inadequate functional integration	Inefficient integration of transplanted stem cells into the existing cardiac tissue, limiting functional integration and electrical coupling (imperfect gap junctions).	Use of tissue engineering approaches to create bio-mimetic cardiac patches or constructs that facilitate the integration of stem cells with the host tissue; genetic modification of stem cells to express cardiac-specific proteins that promote integration and functional coupling; and, evaluation of electrical stimulation techniques to enhance the electrical integration of transplanted stem cells by avoiding imperfect gap junctions.
Lack of standardized protocols	Absence of universally accepted protocols for stem cell preparation, characterization, and delivery in clinical settings.	Establishment of international collaborations and regulatory guidelines to harmonize practices and facilitate the translation of stem cell therapy into clinical applications.
High cost and complexity of manufacturing	Stem cell therapy approaches can be costly and complex in clinical practice.	Streamlining manufacturing processes and scaling up production to reduce costs.
Lack of long-term safety data	The Limited availability of long-term safety data for stem cell therapy, particularly regarding potential adverse and off-target effects.	Conducting long-term safety studies to establish safety profiles and potential long-term complications of stem cell therapy; collaborating among research institutions and regulatory bodies to establish comprehensive safety monitoring and reporting systems.
Limited understanding of therapeutic mechanisms	Incomplete knowledge about the precise mechanisms underlying the therapeutic effects of stem cell therapy in cardiac tissue regeneration.	Conducting more research to unravel the molecular and cellular mechanisms; integration of multi-omics approaches, such as transcriptomics and proteomics, to comprehensively study the changes induced by stem cell therapy in the cardiac tissue microenvironment; and, collaboration between researchers and clinicians to bridge the gap between experimental findings and clinical applications.

The first challenge is the identification of optimal cell types and sources along with the stem cell-based therapy optimization protocols

MSCs, CPCs, and variants of iPSC cells are among the greatest possibilities for stem cell sources [89,90]. Optimization techniques are necessary to boost stem cell retention and tolerance to disease conditions [91]. The combination of gene transfer and stem cell therapy is being explored in preclinical investigations with the possibility of eventual clinical trials [92]. Complementary molecules (mostly anti-inflammatory and anti-apoptotic drugs) have been explored in animal models with promising findings to improve stem cell viability [93,94]. Nanotechnology integration is unavoidable for future cellular therapy development for effective stem cell imaging and to create cell-supporting scaffolds [95]. The rebuilding of the whole organ, biodegradable scaffolds, and scaffold-free cell sheets has also been described [96]. Table 3summarizes certain optimization aspects of stem cell-based therapy.

**Table 3:** A Concise Overview of Various Optimization Protocols of Stem Cell Therapy MI: myocardial infarction

Aspect to optimize	Description
Selection of appropriate stem cell types	Consider differentiation potential, survival rate, immunomodulatory properties, and ease of isolation of stem cells.
Administration routes	Choose appropriate routes such as intramyocardial injection, intracoronary infusion, or intravenous injection for targeted delivery.
Cell dosage and timing	Determine optimal cell dosage based on the size of the injured area and consider timing during the acute or chronic phase after MI.
Supportive strategies	Utilize biomaterials, genetic modifications, and preconditioning techniques to enhance cell survival and therapeutic properties.
Safety considerations	Implement long-term safety monitoring, quality control measures, and close patient monitoring to assess treatment efficacy and safety profiles.
Cell delivery techniques	Develop advanced cell delivery techniques such as cell encapsulation and scaffold systems to improve cell retention, survival, and integration.
Inflammatory response	Modulate immune response through anti-inflammatory strategies and immunomodulatory approaches to promote cell survival and integration.
Microenvironmental factors	Optimize oxygen and nutrient supply and manipulate the extracellular matrix and cytokine environment to enhance stem cell differentiation.
Nanotechnology integration	Harness nanomaterials, nanocarriers, and nanoscale delivery systems to enhance stem cell therapy efficacy, targeted delivery, and tissue regeneration.

The second challenge is the development of novel delivery methods

Developing novel delivery methods for efficient and targeted delivery of stem cells and bioactive factors to the injured heart has been a key area of research in cardiac tissue regeneration.

Biocompatible scaffolds have been developed to provide structural support and augment cellular retention and integration in damaged heart tissue. These scaffolds can be seeded with stem cells and growth factors, allowing for controlled and sustained release of therapeutic agents [97,98]. Injectable hydrogels offer a minimally invasive approach for delivering stem cells and bioactive molecules to infarcted heart tissue. These hydrogels can form a three-dimensional matrix that encapsulates the cells and protects them from harsh mechanical and chemical environments, promoting cell survival and integration [99,100]. Micro- and nanoparticles have gained attention as achieve to facilitate the targeted administration of stem cells to the injured myocardium. These particles can be functionalized to improve cell uptake, provide sustained release, and enable specific targeting of the damaged tissue [101]. Magnetic targeting utilizes magnetic nanoparticles and external magnetic fields to guide and concentrate the cells in the desired anatomical region within the myocardium. This approach enhances the recruitment and retention of the cells, increasing their therapeutic efficacy. Various gene delivery systems have been investigated to augment the regenerative capacity of CSCs. These systems rely on the delivery of genes encoding specific factors to promote stem cell transformation, angiogenesis, and tissue repair. Microbial vectors and genome editing technologies such as CRISPR-Cas9 have been investigated for efficient gene delivery [102]. Finally, EVs, including exosomes, have emerged as a promising direct stem cell delivery alternative. These vesicles can be isolated from stem cell cultures and contain various bioactive molecules such as proteins, nucleic acids, and miRNAs. They can be administered directly or loaded into delivery vehicles to enhance their stability and targeted delivery to damaged heart tissue [103]. Table 4summarizes novel stem cell delivery methods.

**Table 4:** Novel Delivery Methods of Stem Cell Therapy for Ischemic Myocardial Tissue Regeneration and Repair

Novel Delivery Method	Description
Biocompatible Scaffolds	These provide structural support, enhance cell retention, and enable the controlled release of therapeutic agents.
Injectable Hydrogels	Hydrogels form a three-dimensional matrix, protect cells, and promote cell survival and integration in infarcted heart tissue.
Micro- and Nanoparticles	Functionalized particles for targeted delivery of stem cells and therapeutic agents, improving cell uptake and tissue specificity.
Magnetic Targeting	Magnetic nanoparticles and external magnetic fields guide and concentrate stem cells, enhancing their homing and therapeutic efficacy.
Gene Delivery Systems	Delivery of genes encoding specific factors to enhance stem cell differentiation, angiogenesis, and tissue repair.
Extracellular Vesicles	Isolated from stem cell cultures, these vesicles contain bioactive molecules and can be directly administered or loaded into vehicles.

The third challenge is the identification of biomarkers for patient selection and monitoring

Research is focused on identifying biomarkers that can reflect the extent of cardiac damage, predict the regenerative potential of tissues, and provide insights into patient-specific characteristics. Biomarkers specific to cardiac tissue regeneration include myocardial troponins, myosin light chain, brain natriuretic peptide, and various miRNAs [104]. Biomarkers such as interleukins (IL-6, IL-10), c-reactive protein, tumor necrosis factor-alpha, and subsets of immune cells (macrophages, T-cells) can provide insights into the immune modulation during regeneration [105]. Genetic markers, including single nucleotide polymorphisms, and epigenetic markers, such as DNA methylation patterns and histone modifications, can help predict patient outcomes and guide personalized treatment approaches [106]. Non-invasive techniques like imaging (cardiac magnetic resonance imaging and echocardiography) and circulating biomarkers (e.g., miRNAs, circulating endothelial cells) offer the advantage of real-time monitoring and assessment of cardiac tissue regeneration without invasive procedures [107].

Future directions and the ethical dilemma

Scientists of this era are actively investigating alternative stem cell sources to overcome the limitations of traditional sources like ESCs and ASCs. As research progresses, new stem cell sources, tissue engineering approaches, and genetic modifications are being explored to enhance the safety and effectiveness of therapeutics rooted in stem cells. However, addressing the ethical concerns associated with these advancements is crucial.

The creation of ESC lineages is intricately entwined with debates regarding the origins of humanity, and stem cells, being undifferentiated, have the potential to transform into teratomas [108]. A study suggested inhibiting tumorigenic genes in the remaining PSCs as a secondary measure and monitoring diligently to prevent tumor formation [109]. It is important to ensure that the cells are mature and differentiated to reduce the incidence of teratoma formation. However, the risk of teratoma formation remains a concern due to genomic instability and viral vectors, which can induce mutagenesis [110,111]. To address this, effective delivery techniques, molecular strategies, and non-integrating gene delivery methods must be used [15].

Despite the safety issues mentioned above, the current data suggests the widespread generation of cardiac precursor cells from ESCs [112]. Manipulation of somatic stem cells yields iPSCs which can avoid the unique ethical quandaries raised by blastocyst-derived ESCs. iPSCs possess the same features as ESCs, such as regeneration capacity, high telomerase activity, and the ability to differentiate into several subtypes [113]. Regardless of stem cell source, human stem cell research presents difficult ethical quandaries, such as further downstream studies with sensitive implications, approval for making donations for human stem cell research, initial clinical trials involving human stem cell therapies, and oversight of such research activities. It is critical to address ethical and policy issues alongside scientific challenges [114].

MSCs derived from the umbilical cord (UC-MSCs) exhibit myriad advantages compared to their counterparts sourced from alternative origins. These include a non-invasive collection procedure, reduced risk of infection, diminished risk of teratoma formation, ability to differentiate into multiple cell types, and low immunogenicity [115]. The optimal compartment within the umbilical cord for clinical use remains uncertain; however, the era of utilizing UC-MSCs in clinical applications is rapidly approaching.

In forthcoming times, the amalgamation of stem cells with tissue engineering and 3D printing technology offers potential for tissue regeneration and organ transplantation. However, the challenge of generating vascularized tissues compatible with the recipient remains a hurdle within the realm of ischemic myocardial tissue engineering.

Ischemic myocardial tissue engineering has been spotlighted due to poor cell engraftment in cardiac repair. Biomaterials like collagen, fibrin, elastin, and amniotic membrane can be scaffolds to improve reprogramming efficiency. These scaffolds not only provide mechanical support but also provide nutrition and oxygen to the encapsulated cells. An inherent drawback of synthetic biomaterials lies in their inflexibility to accommodate the dynamic movement of cardiac, thereby predisposing them to elicit an inflammatory response [116].

3D bioprinted cardiac patches are another promising approach that helps regenerate cardiac tissue and can be used therapeutically [117]. Noor et al. have made a noteworthy accomplishment in tissue engineering by employing 3D bioprinting. The researchers have produced a cellularized human heart, which includes significant blood vessels, through the utilization of hydrogels containing cardiac and endothelial cells [118]. Therefore, it is necessary to develop methodologies that facilitate the visualization of the entire cardiovascular network and its incorporation into the structural design of the organ. Advanced technologies that precisely print these slender blood vessels within intricate anatomical structures are paramount.

Lastly, advancements in genetic engineering techniques, such as CRISPR-Cas9, provide opportunities for precise genetic modifications in stem cells [119]. Genetic modifications can enhance stem cell survival, improve their therapeutic properties, and promote targeted tissue regeneration in the cardiac context [120]. Transplantation of such genetically modified cells may prevent inflammatory reactions in the host, immune rejection, hypoxia, and apoptosis [121]. However, ethical concerns arise regarding potential unintended consequences, off-target effects, and hereditary implications [122]. A thorough evaluation of the ethical implications and long-term effects of genetic modifications should be conducted to ensure the responsible and ethical use of these techniques. Other ethical considerations to note are summarized in Table 5below.

**Table 5:** Ethical Considerations for Stem Cell-Based Therapy

Ethical Considerations	Description
Informed consent	Informed consent processes must be implemented to provide patients participating in clinical trials with comprehensive information about the treatment, potential risks, benefits, and alternatives.
Patient privacy and confidentiality	Protecting patient privacy and confidentiality is an ethical imperative in stem cell research. Adhering to ethical guidelines and implementing secure data management systems are essential to safeguard patient information.
Safety and efficacy	Rigorous evaluation of safety and efficacy through preclinical and clinical studies is essential. Ethical guidelines should emphasize evidence-based practices and transparent reporting of results.
Access and equity	Ethical considerations should ensure equitable access to stem cell therapies, addressing affordability, availability, and fair distribution to prevent exacerbating healthcare disparities.
Responsible research and regulation	Conducting stem cell research with integrity and transparency is an ethical obligation. Researchers should adhere to ethical guidelines, promote reproducibility, and ensure the responsible use of stem cell resources.
Public perception and education	Educating the public about stem cell therapy, its potential benefits, and its limitations is an ethical responsibility. Transparent communication, addressing misconceptions, and fostering public trust are essential for the ethical implementation of stem cell therapies.

Regulatory challenges in stem cell-based therapy

Ensuring the safety and efficacy of stem cell therapy is a critical regulatory challenge. Robust preclinical and clinical trials are necessary to demonstrate the therapeutical benefits and assess conceivable hazards. These trials should follow rigorous study designs, incorporate appropriate endpoints, and include prolonged surveillance to appraise the enduring effects of such interventions [123]. Another challenge is establishing standardized protocols and quality control measures for stem cell production, characterization, and delivery. Regulatory agencies require stringent oversight to ensure consistent and reliable therapies [124]. One major challenge is to develop a scalable manufacturing process that can reliably produce high-quality stem cell products. Implementing good manufacturing practices and complying with regulatory guidelines are essential to ensure the reproducibility and safety of stem cell therapies [125,126]. Finally, addressing patient safety is a regulatory challenge in stem cell therapy. Developing appropriate patent protection mechanisms and navigating licensing agreements can facilitate the translation of stem cell therapies to clinical practice [127].

## Conclusions

While challenges remain to be addressed, the prospective outlook for stem cell therapy in the domain of ischemic tissue repair and regeneration holds great promise. A wide array of stem cells have demonstrated favorable outcomes through several mechanisms, including angiogenesis, paracrine signaling, and the like. Optimization protocols such as novel cell delivery methods, timing, dosage, and co-administration of supportive agents are vital in maximizing cell survival, differentiation, integration, and immune response modulation. New stem cell sources like iPSCs and MSCs provide alternative options for overcoming ethical and immune-related challenges. Tissue engineering approaches, including scaffold-based strategies and 3D bioprinting, offer innovative ways to create functional cardiac tissue constructs-genetic modifications, including CRISPR-Cas9 technology, open avenues for enhancing stem cells' therapeutic potential. Nanotechnology has the potential to further improve stem cell therapy by enabling targeted delivery of growth factors. These advancements pave the way for personalized, targeted, and effective stem cell-based therapies in cardiac regeneration. Despite these advancements, the future of utilizing tissue-specific progenitor stem cell populations for treating organ-specific diseases remains a mystery.

Robust regulatory frameworks, including appropriate preclinical and clinical trial design, and standardized manufacturing processes, are essential for ensuring the safety and efficacy of stem cell-based therapies. Intellectual property rights and commercialization strategies also significantly incentivize further research and development. Responsible research, transparent communication, and a striking balance between scientific progress and ethical principles are essential for successfully translating stem cell therapies into clinical applications while upholding ethical standards in this rapidly evolving field.
